# A Comparative Study of Connective Tissue Metabolism Indices in Experimental Comorbidity-Free Periodontitis and Periodontitis Combined with Thyroid Dysfunction

**DOI:** 10.25122/jml-2019-0113

**Published:** 2020

**Authors:** Vitaliy Shcherba, Mariia Kyryliv, Iryna Bekus, Inna Krynytska, Mariya Marushchak, Mykhaylo Korda

**Affiliations:** 1. Department of Dentistry, I. Horbachevsky Ternopil National Medical University, Ternopil, Ukraine;; 2. Department of General Chemistry, I. Horbachevsky Ternopil National Medical University, Ternopil, Ukraine;; 3. Department of Functional and Laboratory Diagnostics, I. Horbachevsky Ternopil National Medical University, Ternopil, Ukraine;; 4. Department of Medical Biochemistry, I. Horbachevsky Ternopil National Medical University, Ternopil, Ukraine

**Keywords:** Connective tissue, periodontitis, thyroid dysfunction

## Abstract

Periodontal disease is a chronic bacterial infection characterized by persistent inflammation, connective tissue breakdown, and alveolar bone destruction. The current study aimed to compare the connective tissue metabolism indices in rats with comorbidity-free periodontitis and in animals with periodontitis in a setting of hyper-and hypothyroidism. 12-14-week-old inbred white male rats (n=48) were included in the experiment. They were randomly divided into the following groups: control, animals with a model of periodontitis, animals with periodontitis in a setting of hyperthyroidism, animals with periodontitis in a setting of hypothyroidism. Serum levels of free thyroxine, free triiodothyronine, and thyroid-stimulating hormone were assayed using ELISA kits manufactured by Vector Best (Russia) to confirm the hyper- and hypothyroid status. Collagenolytic activity, the content of glycosaminoglycans, free hydroxyproline, and fucose, unbound with proteins in blood serum were assayed using the spectrophotometric method. We have found the increasing of collagenolytic activity by 46.1% (р<0.001), the content of free hydroxyproline by 74.1% (р<0.001), the content of glycosaminoglycans by 1.8 times (р<0.001), the content of fucose, unbound with proteins by 2.8 times (р<0.001) in rats with periodontitis vs. the control group. The development of periodontitis in a setting of thyroid dysfunction leads to an even more significant increase in the destruction of connective tissue, which is confirmed by a significant increase in the content of studied indices vs. euthyroid animals, both in hyperthyroidism and hypothyroidism.

## Introduction

Periodontitis is a chronic non-communicable disease (NCD) that shares social determinants and risk factors with the major NCDs that cause around two-thirds of deaths, such as heart disease, diabetes, cancer, and chronic respiratory disease [1]. The Global Burden of Disease Study indicates that severe periodontitis is the 6th most prevalent disease worldwide, with an overall prevalence of 11.2% and around 743 million people affected [2]. Overall, it affects about 20-50% of the population around the globe [3] and is the most common oral condition of the human population [4]. In Europe, epidemiological evidence indicates that mild gingival inflammation and mild to moderate loss of periodontal attachment in specific sites are prevalent in the adult population, but epidemiological studies available from most Eastern European countries are limited [5].

The periodontium is a unique organ that consists of two soft connective tissues (gingival and periodontal ligament) and two calcified components (cementum and alveolar bone) [6]. The extracellular matrix (ECM) of the connective tissue (CT) is represented by fibrous structures as well as by a gel-forming medium formed by glycosaminoglycans (GAGs) [7]. Sulfated glycosaminoglycans (GAGs) - chondroitin-4-sulfate, chondroitin-6-sulfate, dermatan sulfate, heparan sulfate, and keratan sulfate provide stabilization and cementation of fibrous structures, protect cells from the penetration of microorganisms and their toxins, regulate water-salt metabolism in tissues, participate in intercellular signaling and regulate the activity of growth factors, including the fibroblast growth factor [8, 9].

Periodontal disease is a chronic bacterial infection characterized by persistent inflammation, connective tissue breakdown, and alveolar bone destruction [10, 11]. The aggression of periodontopathogenic microflora is associated with the presence in the shell of bacteria of proteolytic enzymes and endotoxins. Microbial enzymes (hyaluronidase, chondroitin sulfatase, protease, glucuronidase, collagenase) cause the depolymerization of proteoglycans and GAGs of the periodontium and disruption of their resynthesis, resulting in endotoxin invasion of tissue [12]. Therefore, the depolymerization of CT biopolymers is an essential link in the pathogenesis of the periodontal disease.

Metabolic processes occurring in CT and its remodeling are largely determined by the functional state of hormonal systems [13], including thyroid hormones.

Therefore, this paper aims to compare the connective tissue metabolism indices in rats with comorbidity-free periodontitis and in animals with periodontitis in a setting of hyper-and hypothyroidism.

## Material and Methods

### Animals

12-14-week-old inbred white male rats (n=48) with a bodyweight of 180-200 g were included in the experiment. The animals were kept under standardized conditions, with controlled light cycle (12/12) and unlimited access to water and food throughout the period of the experiment.

### Study groups

The animals were randomly divided into the following groups: Group І: control animals were administered intragastric 1% starch solution (n=12); Group ІІ: animals with a model of periodontitis (n=12). During two weeks, the rats in this group were administered 40 μL (1 mg/mL) of E. coli lipopolysaccharide (LPS) (manufactured by Sigma-Aldrich, USA) into gingival tissues every other day [14]. Group ІІІ: rats with periodontitis in a setting of hyperthyroidism (n=12). To create an experimental model of thyroid hyperfunction, the animals received daily intragastric doses of L-thyroxine in a 1% starch solution at 10 μg/day per 100 g of body weight for 21 days [15]. Starting from day 8 of the experiment, the rats were given LPS into gingival tissue for two weeks; Group IV included rats with periodontitis in a setting of hypothyroidism (n=12). To create an experimental model of thyroid hypofunction [15], the animals received daily intragastric doses of methimazole in a 1% starch solution at 1 mg/day per 100 g of body weight for 21 days. The rats were given LPS into the gingival tissue for two weeks starting from day 8 of the experiment.

The rats were euthanized under deep thiopental-sodium anesthesia on day 22 from the onset of the experiment. Blood serum was used for further investigation.

All manipulations with experimental animals were performed according to provisions of the European Convention for the Protection of Vertebrate Animals used for Experimental and other Scientific Purposes [16]. The Bioethics Commission of I. Horbachevsky Ternopil National Medical University, Ternopil, Ukraine approved the protocol of the experiment (Excerpts from Minutes No. 59, dated 23.10.2019).

Serum levels of free thyroxine (FT4), free triiodothyronine (FT3), and thyroid-stimulating hormone (TSH) were assayed with ELISA kits manufactured by Vector Best (Russia) yo confirm hyper- and hypothyroid status.

Collagenolytic activity, the content of glycosaminoglycans and fucose, unbound with proteins, were determined using the methods of P.N. Sharaev and co-authors [17-19]; the content of free hydroxyproline was determined using the method of S.S. Tetyanets [20].

Statistical processing of digital data was carried out using the software Excel (Microsoft, USA) and STATISTICA 6.0 (Statsoft, USA). The distribution of data was analyzed according to the assessment of normality by the Kolmogorov-Smirnov criterion. The obtained values had a normal distribution, so the difference between the groups was analyzed using the Student’s t-criterion. All data were presented as M (mean) ± m (standard error). A probability level (p-value) of less than 0.05 was considered to be statistically significant.

## Results

The analysis of data indicated that collagenolytic activity in the blood serum of rats with periodontitis was increased by 46.1% (р<0.001) vs. control group (Table 1). In rats with periodontitis in a setting of hyperthyroidism, this index has increased by 2.3 times (р<0.001) vs. control group. It should be noted that the collagenolytic activity in the blood serum of hyperthyroid rats significantly exceeded this index (by 55.6%) in rats with periodontitis without concomitant pathology and by 17.2% (p<0.01) in rats with periodontitis in a setting of hypothyroidism.

**Table 1:** The indices of connective tissue metabolism in blood serum of rats with periodontitis without comorbidities and in a setting of hyper-and hypothyroidism (M ± m, n=12).

**Index**	**Group of animals**			
	**Control**	**Periodontitis**	**Periodontitis in a setting of hyperthyroidism**	**Periodontitis in a setting of hypothyroidism**
**Collagenolytic activity,** **μmol/l×hour**	5.40 ± 0.18	7.89 ± 0.45 p_1_<0.001	12.28 ± 0.38 р_1_<0.001 p_2_<0.001	10.48 ± 0.33 р_1_<0.001 p_3_<0.001 p_4_<0.01
**Free hydroxyproline,** **μmol/l**	11.9 5± 0.33	20.80 ± 0.76 p_1_<0.001	30.20 ± 0.80 р_1_<0.001 p_2_<0.001	25.21 ± 0.55 р_1_<0.001 p_3_<0.001 p_4_<0.001
**Glycosaminoglycans, μmol/l**	42.38 ± 1.65	77.81 ± 2.29 p_1_<0.001	109.83 ± 4.97 р_1_<0.001 p_2_<0.001	97.85 ± 3.45 р_1_<0.001 p_3_<0.001 p_4_>0.05
**Fucose, unbound with proteins, μmol/l**	79.93 ± 3.28	227.39 ± 10.70 p_1_<0.001	389.55 ± 15.40 р_1_<0.001 p_2_<0.001	304.78 ± 9.63 р_1_<0.001 p_3_<0.001 p_4_<0.001

Note: р1 – significant differences compared to control animals; р2 – significant differences between the group of periodontitis with the group of periodontitis combined with hyperthyroidism; р3 – significant differences between the group of periodontitis with the group of periodontitis combined with hypothyroidism; р4 – significant differences between the group of periodontitis combined with hyperthyroidism with the group of periodontitis combined with hypothyroidism.

In rats with a model of periodontitis in a setting of hypothyroidism, the collagenolytic activity increased by 1.9 times (p<0.001) vs. control group and significantly exceeded the index of animals with periodontitis without concomitant pathology by 32.8% (p<0.001).

Activation of collagenolysis is also evidenced by an increased content of free hydroxyproline. Thus, this index in blood serum of rats with periodontitis increased by 74.1% (р<0.001), in rats with periodontitis in a setting of hyperthyroidism by 2.5 times (р<0.001), in rats with periodontitis in a setting of hypothyroidism by 2.1 times (р<0.001) vs. control group. It should be noted that the content of free hydroxyproline in blood serum of hyperthyroid rats significantly exceeded this index (by 45.2%) in rats with periodontitis without concomitant pathology and by 19.8% (p<0.001) in rats with periodontitis in a setting of hypothyroidism.

In rats with a model of periodontitis in a setting of hypothyroidism, the content of free hydroxyproline in blood serum significantly exceeded the data of rats with periodontitis without concomitant pathology by 21.2% (p<0.001).

Glycoproteins and proteoglycans are the major proteins in the periodontal connective tissue. The content of glycosaminoglycans (GAGs) in blood serum can be considered as a biochemical marker of proteoglycans decomposition [12].

The analysis of data indicated that the modeling of periodontitis in rats has led to the increased content of GAGs in blood serum by 1.8 times (р<0.001) vs. control group. In rats with periodontitis in a setting of hyperthyroidism, this index has probably increased by 2.6 times (р<0.001) vs. control group. It should be noted that the content of GAGs in blood serum of hyperthyroid rats significantly exceeded this index (by 41.2%) in rats with periodontitis without concomitant pathology.

In the case of periodontitis in a setting of hypothyroidism, this index significantly increased by 2.3 times vs. control group and by 25.8% (p<0.001) exceeded this index in rats with periodontitis without concomitant pathology. In the meantime, no significant differences were found between the animals with periodontitis in a setting of hyper-and hypothyroidism.

The degree of glycoproteins destruction was evaluated by the content of fucose, unbound with proteins. It has been established, that content of fucose, unbound with proteins in blood serum of rats with periodontitis increased by 2.8 times (р<0.001), in rats with periodontitis in a setting of hyperthyroidism – by 4.9 times (р<0.001), in rats with periodontitis in a setting of hypothyroidism by 3.8 times (р<0.001) vs. control group. It should be noted that the content of fucose, unbound with proteins in blood serum of hyperthyroid rats, significantly exceeded this index (by 71.3%) in rats with periodontitis without concomitant pathology and by 27.8% (p<0.001) in rats with periodontitis in a setting of hypothyroidism. In the case of periodontitis in a setting of hypothyroidism, this index significantly exceeded this index (by 34.0%) in rats with periodontitis without concomitant pathology.

## Discussion

Our research has found that in rats with periodontitis, collagenolytic activity, and content of free hydroxyproline in blood serum increased. Collagen is the main structural protein of the periodontal intracellular matrix of CT. An enzyme collagenase, which is synthesized by CT cells (fibroblasts and macrophages) and is found in four isoforms, regulates collagen metabolism. Collagenase activity depends on the ratio in the intracellular matrix of its activators and inhibitors. Plasmin, kallikrein, and cathepsin B play a special role in its activation in inflammatory processes [21].

Activation of collagenolysis in experimental periodontitis reflects an increase in catabolic processes in the CT structures of the periodontium, which contribute to the violation of its supporting function, characterized by a decrease in the content of collagen in periodontal tissues [22].

The increase of catabolic processes in the CT of the periodontium in the case of experimental periodontitis was also confirmed by the increase of glycoproteins degradation marker (the content of fucose, unbound with proteins) and an increase of GAGs content (proteoglycans degradation marker). This leads to the disorganization of not only the collagen structures of the CT but also the depolymerization of the organic matrix components and disruption of their resynthesis.

The depolymerization of CT biopolymers and their impaired resynthesis is an important link in the pathogenesis of inflammatory and dystrophic periodontal diseases. On the one hand, this is facilitated by the production of exotoxins and histolytic enzymes (hyaluronidases, chondroitin sulfatases, proteases, glucuronidases, collagenases) by pathogenic microorganisms, which causes depolymerization of collagen, proteoglycans, and glycoproteins. On the other hand, CT destruction is associated with endogenous activation of matrix metalloproteinases (MMPs), plasmin, serine proteinases of polymorphic nuclear leukocytes, and their phagocytic activity in response to the production of proinflammatory cytokines [8, 23, 24].

The destruction of CT biopolymers can also be associated with the development of oxidative stress in periodontal tissues, which causes oxidative modification of proteins and carbohydrates, apoptotic changes, or induces the production of histolytic enzymes due to the activation of redox-sensitive transcription factors (NF-κB) [25-27]. NF-κB-dependent processes associated with the activator of the NF-κB receptor (RANK), its ligand (RANKL), and the false receptor osteoprotegerin are important regulators of the resorptive activity of osteoclasts [28].

The development of periodontitis in a setting of thyroid dysfunction leads to an even greater increase in the destruction of CT, which is confirmed by the significant predominance of the catabolic indices vs. euthyroid animals, both under hyperthyroidism and hypothyroidism. It should be noted that when comparing CT catabolism indices in hyperthyroid and hypothyroid rats, higher rates were observed in hyperthyroid animals, except GAGs content.

Several data suggest that the thyroid gland has a regulatory influence on bone and connective tissue metabolism [29-31]. Triiodothyronine (T3) stimulates osteoblast proliferation, differentiation and apoptosis, and increases the expression of osteocalcin, type 1 collagen, alkaline phosphatase, metalloproteins, IGF-1, and its receptor (IGF-1R). Subsequently, during bone resorption, T3 increases the expression of important differentiation factors of the osteoclast lineage, such as interleukin (IL) 6 and prostaglandin E2. It has also been demonstrated that T3 increases the expression of mRNA of the ligand of receptor activator of nuclear factor-κβ (RANKL) in the osteoblast, which activates RANK present in osteoclast precursors, a key step in osteoclastogenesis [32]. Ziegelhoffer-Mihalovicova B. et al. also showed that triiodothyronine injections increase the mRNA level for both procollagen type I and III in the rat heart after 72 hours [33].

T4 may also affect bone remodeling, enhancing osteoclastic activity by stimulating prostaglandin secretion [34]. Thyroxin was proved to reduce the high level of hyaluronic acid (HA) in the skin of patients with myxoedema [35].

The expression of the TSH receptor (TSHR) has been demonstrated in osteoblasts and osteoclasts, suggesting that TSH may have direct effects on these cells [32].

We assume that thyroid dysfunction exacerbates CT catabolism due to the hyperproduction of proinflammatory cytokines. There are data suggesting that progressive destruction of bone and connective tissue in patients with periodontitis is associated with a combination of cytokines - interleukins (IL) 1β, 6, tumor necrosis factor-α (TNF-α), and prostaglandins E2 [36]. Inflammatory cytokines spread into periodontal tissues by entering the systemic circulation where they induce the production of matrix metalloproteinases, responsible for connective tissue and further alveolar bone destruction by activation of the resident cells of periodontium [37]. Monea et al. suggested that cytokines IL-6 and TNF-α produced in thyroid disorders play a major role in the initiation and amplification of the inflammatory cascade in the periodontal tissues. The endotoxins produced by the bacteria in dental plaque combines with these cytokines, further aggravating the inflammatory cascade by the production of more cytokines responsible for MMPs activation and periodontal breakdown [38]. Babior B.M. suggested that polymorphonuclear leukocytes (PMNs) play a major role in bacterial phagocytosis by the respiratory burst mechanism through the nicotinamide adenine dinucleotide phosphate-oxidase complex and leads to the production of reactive oxygen species (ROS) which generates oxidative stress within periodontal tissues. These ROS lead to bone resorption by acting at the ruffled border of osteoclasts [39]. Similarly, Mezosi E. et al. have shown that thyroid hormones stimulate free radical production and impaired phagocytosis by PMNs, mainly in hypothyroid patients [40]. Kanatani M. et al. showed a negative effect of thyroid dysfunction on IL-6 and TNF-α, which are responsible for osteoclast differentiation and function independent of the RANKL mechanism [41].

## Conclusion

Our studies showed the increase of connective tissue catabolism in rats with periodontitis, as evidenced by an increase in collagenolytic activity, in the free oxyproline content, in the proteoglycans degradation marker – the content of glycosaminoglycans, in the glycoproteins degradation marker – the content of fucose, unbound with proteins.

The development of periodontitis in a setting of thyroid dysfunction leads to an even greater increase in the destruction of connective tissue, which is confirmed by a significant increase in the content of connective tissue catabolism indices vs. euthyroid animals, both in hyperthyroidism and hypothyroidism.

## Conflict of Interest

The authors declare that there is no conflict of interest.
